# Ethnobotanical study of medicinal plants used by the indigenous community of the western region of Mizoram, India

**DOI:** 10.1186/s13002-023-00642-z

**Published:** 2024-01-03

**Authors:** Laldinfeli Ralte, Hmingremhlua Sailo, Y. Tunginba Singh

**Affiliations:** Department of Botany, Mizoram University, Aizawl, Mizoram 796004 India

**Keywords:** Mizo people, Ethnobotany, Traditional medicine, EthnobotanyR, Medicinal plants, Mizoram

## Abstract

**Background:**

Plants have long been utilized as traditional medicines by the inhabitants. However, until recently, the traditional knowledge had not been extensively documented from the hilly state of Mizoram, India. The present study was designed to perform a quantitative analysis of ethnomedicinal plants used by Mizo tribes using quantitative ethnobotanical indices. The study attempts to find new ethnomedicinal plant species that could be a source for the discovery of new drug formulations.

**Methods:**

The information was obtained through extensive and semi-structured interviews. Quantitative indices such as informant consensus factor (ICF), use value (UV), fidelity level (FL), relative frequency of citation (RFC), and relative importance index (RI) were used to quantify the advantages, significance, and coverage of ethnomedicine. All the collected data were analyzed using the ethnobotanyR package in R.

**Results:**

A total of 124 ethnomedicinal plant species, distributed in 112 genera under 60 families, were documented from 206 informants. Herbs (49.19%) were the most dominant growth form, and leaves (49.19%) were the most common plant parts used for the preparation of herbal medicine while decoction (61.21%) was the most popular formulation. Asteraceae (11) were the most common families among the documented species. Digestive disease, burns, cuts, and wounds had the highest ICF value (0.94), and *Lepionurus sylvestris* had the highest FL (91%). *Oroxylum indicum* (6.25) was the most commonly utilized ethnomedicinal plant based on UV, RI had the highest value in *Blumea lanceolaria* (1.12), and *O. indicum* (0.29) had the highest RFC value. According to the findings, the traditional medicinal plant treatment is still widely used in the research area.

**Conclusion:**

Documentation of new ethnomedicinal species and their therapeutic usage will encourage further phytochemical and pharmacological research, potentially leading to the discovery of new drug formulations.

## Background

Throughout history, plant resources have remained an important aspect of human society. Following the fulfillment of necessities such as food and shelter, man has looked for appropriate medicine among plants to cure ailments [1]. Medicinal plants have been utilized for many years to treat various ailments not only in rural areas but also increasingly in the urban areas of both developed and developing countries [2]. According to the World Health Organization, Global Centre for Traditional Medicine (2023) around 88% of the world’s population relies on herbal medicine for their primary health-care requirements, particularly in rural areas [3]. Due to the dearth of modern health-care systems in developing countries, traditional medicines provide a low-cost source of basic health care [4]. Documentation and research work on these plants have shown to be an effective technique for understanding how diverse indigenous people interact with natural resources, particularly for medical and pharmaceutical purposes [5]. Ethnomedicinal research has aided in the creation of both natural and synthetic medications [6]. Interestingly, ethnobotanical knowledge has been used as an introduction for numerous successful drug screening studies [7]. Likewise, ethnomedicine can be traced back to the origins of more than half of all pharmaceutical medications [7]. However, adequate archiving of such knowledge, particularly traditional ethnomedicinal techniques, is critical since ethnomedicinal healers have a long relationship with herbs and their medicinal characteristics. Ethnomedicinal information is typically passed verbally through families from one generation to the next [8]; hence, the majority of this knowledge has not been systematically documented [9]. Traditional medicinal practices, on the other hand, have been steadily declining in recent years, and no comprehensive investigation on the ethnomedicinal properties has been conducted so far, owing to a lack of interest among the younger generation in traditional treatment systems, mass deforestation, and rural depopulation accelerated loss of valuable traditional knowledge [10].

India’s health-care system varies greatly, serving both urban and rural populations that use modern and traditional medical practices [4]. In addition, enterprises and institutions have their health insurance programs. Due to financial constraints, treating the large population’s illnesses mostly involves the use of traditional knowledge and practices, especially in rural regions, or traditional healers, and Mizoram is one such state in the northeastern region of India. A significant portion of the research sites are remote areas, with poor transportation and electrical infrastructure. Many of the villages lack access to public health-care facilities, which forces residents to rely on traditional medicines.

While several researchers [11–16] have found and recorded various medicinal plants of Mizoram, describing their distribution, preparation mode, uses, and habitat, the majority of their reports have come from the central regions of the metropolis. Their research brought the qualitative facts to light, but no comprehensive ethnobotanical research has been documented in the western region of Mizoram. To determine which plants are most valuable and to record the traditional uses of medicinal plants in the western region of Mizoram, India, the current study intends to conduct a quantitative investigation utilizing various cultural relevance indices. Their established practical knowledge is founded on over a century of observation and credibility. Furthermore, the present study aims to find new ethnomedicinal plant species in the research area, which could be a source for the discovery of new drug formulations.

## Methods

### Study area and site description

Mizoram is one of the eight states in the northeastern region (NE), one of the biodiversity hotspots in the Indian center, which is widely known for its richness in ethnic diversity and traditional culture [17]. The state is an expanse of blue–green hills and covers an area of 21,087 km^2^. It is located between 21° 58′ N and 24° 35′ N latitudes and 92° 16′ E and 93° 29′ E and is flanked by Assam in the north, Manipur in the north–east, Myanmar in the east and south, and the west by Bangladesh and the state of Tripura. The state is largely hilly with deep gorges in between hill ranges that run north–south with an average elevation of 920-m above sea level. The mountain ranges extend north–south, interrupted by narrow deep valleys and crisscrossed by a plethora of little hillocks. Mizoram is home to the Mizo indigenous community, and the vast majority of the population consists of various ethnic tribes that are either culturally or linguistically related. These communities come from various ethnic groups and use traditional ethnomedicine for their primary health-care system [18].

The study was conducted in Mamit district, western region of Mizoram which lies approximately 23.93° N and 92.48° E. It is composed of 100 villages and three tehsils (sub-district/block) with a total land area of 3025.75 km^2^. The study areas are classified as rural, mainly composed of agricultural lands. It is surrounded on the north by the Hailakandi district of Assam, on the west by North Tripura district of Tripura state, and shares an international border with Bangladesh on the north. Jhum cultivation is the most popular and comprises the major source of agricultural products. Forests account for approximately 2774 km^2^ of the district’s total area. The annual average temperature ranges from 9 to 24 °C and from 24 to 36 °C during winter and summer, respectively. Mamit district is also home to Dampa Tiger Reserve. This area has a low mountainous landform type, including Reiek Mountain, which rises to a height of 1070-m above sea level. The average height is 718 m, with the lowest point being 115-m high at the terminus of the Langkaih River (https://mamit.nic.in). The region has a subtropical humid monsoon climate, which is defined by long summers and short winters. The region has been able to retain an abundance of medicinal plant resources due to the complicated terrain, elevation, and altitude differences. These resources assist the local people and their traditional medicine practices.

### The Mizo people and their ethnographic background

According to the 2011 census, the western region of Mamit district has a population of 86,364 which gives it a ranking of 618th in India (out of 640). With 29 people per square kilometer, the district has a low population density, and a majority of the areas are covered with forests. Between 2001 and 2011, its population grew at a pace of 37.56%. A total of 927 females for every 1000 males is the sex ratio, and 84.93% of the population is literate. In urban regions, only 17.25% of people reside, and the scheduled tribes comprise 95.04% of the population. In the western region, the Mizo people account for 62.61% of the total population, and the remaining consists of Tripuri, Chakma, and Bengali. Since the majority of the local people are Mizo, Mizotawng (62.61%) is the major language used by the local people, followed by Bru (17.64%), Chakma (15.25%), and Bengali (1.92%). Prior to the Mizo people’s conversion to Christianity, which began with the British occupation of the area, the majority of the Mizo people practiced the Mizo religion, also known as Lushai animism. According to the 2011 census, Christianity (80.01%) is the major religion among the local people of the western region of Mizoram, followed by Buddhism (14.27%), Hinduism (3.46%), Islam (2.06%), and others (0.20%). Generations of the Mizo people have flourished in the western region due to the dense forests and temperate environment. However, modern medical supplies are frequently short because of transportation constraints. The Mizo people have a great deal of expertise in using native plants which they refer to as “ramhmul damdawi,” to prevent and treat a wide range of diseases because of their prolonged struggles with illness. People in the Mizo community frequently know a variety of medicinal plants, which has led to the accumulation of a large number of prescriptions for the prevention and treatment of illness.

The Mizo people rely on a variety of subsistence pursuits including farming, hunting, and tending to agricultural plots. The indigenous people are mostly farmers and engage in small-scale migratory slash-and-burn agriculture that follows the rainforest’s seasonal cycles. They engage in four primary crop systems such as swidden, slash and burn, and agriculture, which uses short-cycle crops such as rice (*Oryza sativa* L.), corn (*Zea mays* L.), and pumpkin (*Cucurbita maxima* Duchesne) which are cultivated in regrown or virgin forests, agriculture, a distinctive activity among the indigenous people, with plantations of rubber tree (*Hevea brasiliensis* (Willd. ex A. Juss.) Mull. Arg.), areca nut (*Areca catechu* L.), and banana (*Musa* spp.) which are the major crops, and home gardens mainly grown aromatic and medicinal plants (https://mizoram.nic.in). Historically, all of the Mizo people’s uses and consumptions were derived from locally accessible resources and were collected or produced locally.

### Data collection and identification of medicinal plants

**Fig. 1:**
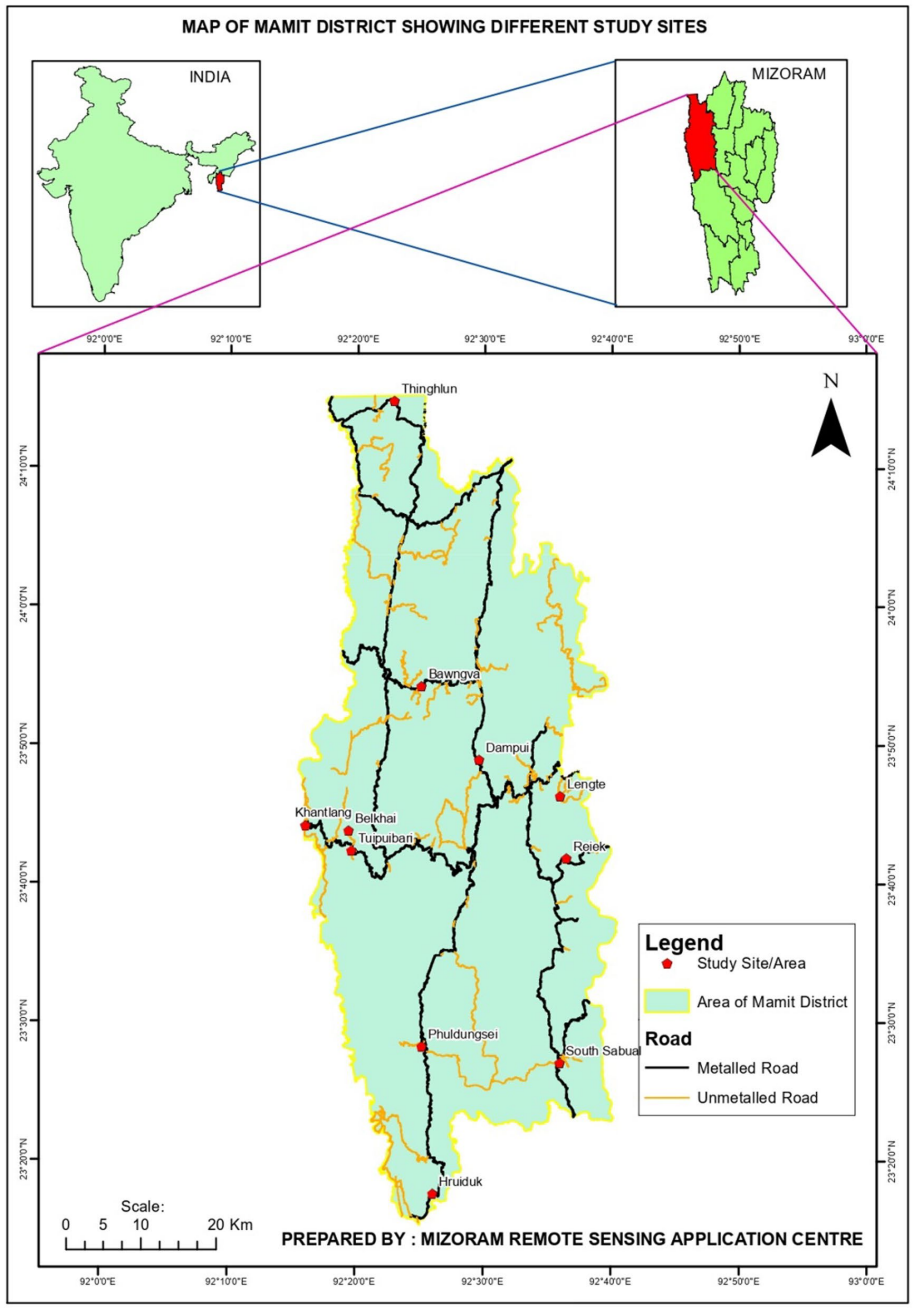
The study area for documentation of ethnomedicinal plants and collection of plant species, Mamit district, Mizoram, India (Photo courtesy: Mizoram Remote Sensing Application Centre, Government of Mizoram)

**Fig. 2:**
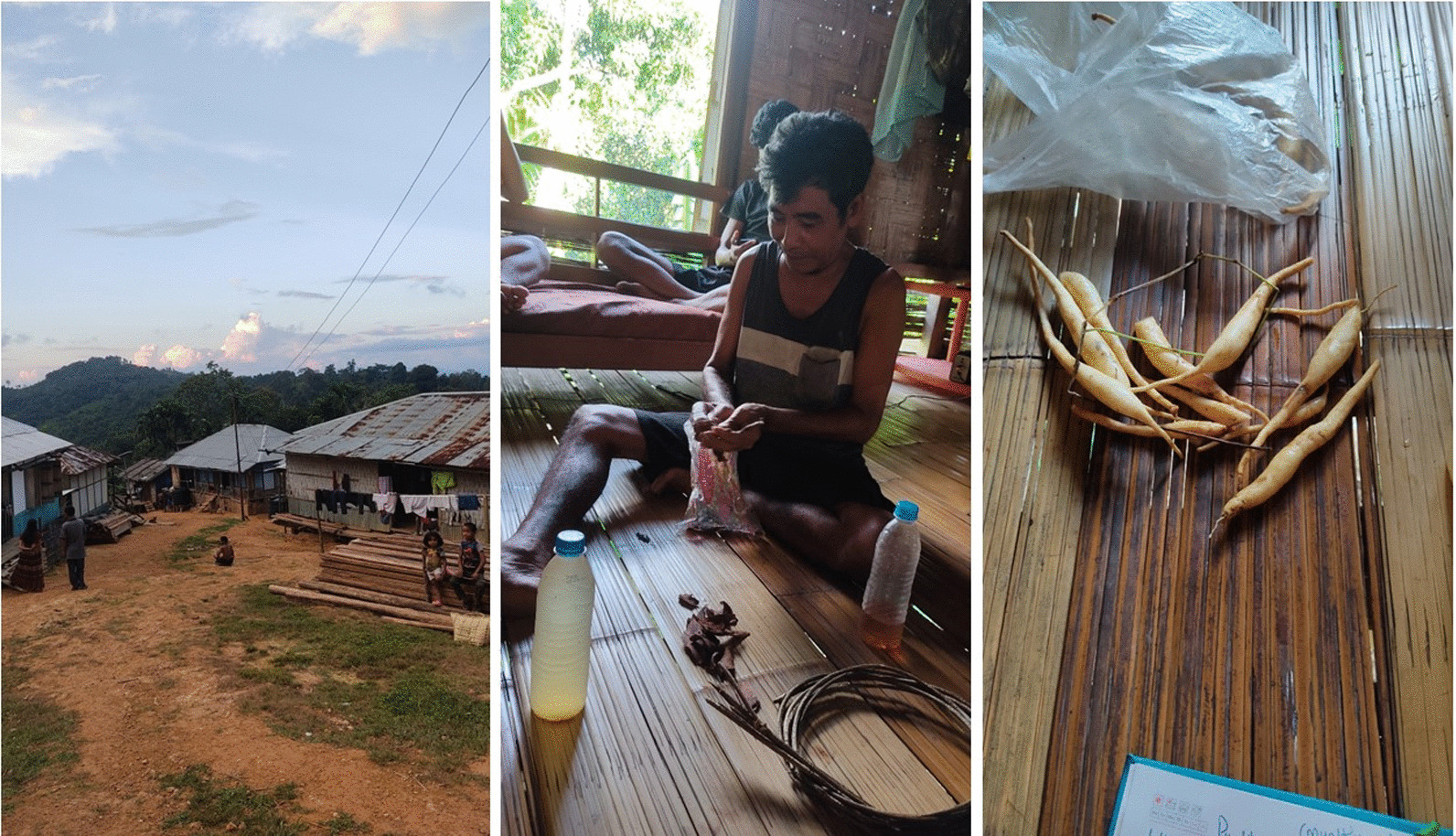
Photograph of the study area, informant, and medicinal plant specimen

Ethnobotanical data were collected from August to November 2021 in 11 villages of Mamit district, Belkhai, Bawngva, Dampui, Hruiduk, Phuldungsei, Lengte, Khantlang, Reiek, South Sabual, Thinghlun, and Tuibuibari (Fig. 1). Before starting the study, informed consent was obtained. To acquire information on the therapeutic plants utilized by the indigenous people, unstructured and semi-structured interviews were conducted and prepared questionnaire that had been ethically evaluated and approved by the Institution Human Ethical Committee (IHEC) of Mizoram University. A total of 206 informants, including 127 males and 79 females, were interviewed. Fifty-nine key informants were traditional healers skilled in traditional medicine, using a purposive technique among the 206 informants. The remaining 147 informants, in contrast, lacked specialized knowledge and were either traditional medicine users or information producers. The age of the informants ranged between 26 and 87 years. Of the 206 informants, 22.82% of them were between 60 and 87 years of age. No discussion of confidential remedies was included in the study to safeguard the informants’ intellectual property rights. Each person gave their verbal agreement before the interview. To acquire information about the therapeutic plants used by the local people, we used semi-structured and unstructured interviews. The demographic information of the informants such as age, educational level, and gender, along with the local name of the plants used for medication, form of administration, mode of preparation, and plant parts used was also documented. Medicinal plant specimens were collected with the help of informants from the available areas (Fig. 2). Three to five branches of medicinal plants, preferably with reproductive parts such as flowers and fruits, were used to make herbarium specimens. Identification of the medicinal plants was done by Dr. Kh. Sandhyarani Devi, Taxonomist, Department of Botany, Mizoram University, and online databases such as World Flora Online [19] and International Plant Names Index [20]. The voucher specimens were collected and deposited in the herbarium of the Department of Botany, Mizoram University, for future study and reference.

### Data analyses

Using MS Excel, the gathered data were revised and organized following use reports. The voucher number, scientific and local names, habit, part used, mode of preparation, and medicinal uses are included in each column as attributes of that reference. SPSS software was used for statistical analyses.

The ethnobotanyR package in R was used to perform quantitative analysis [21]. The chord diagram of plant part usage and informant consensus factor were plotted using ethnobotanyR package in R software. To meet the requirements of ethnobotanyR, data were specifically organized and prepared in a particular data frame. According to informant consensus, common quantitative ethnobotany indices were calculated using the ethnobotanyR program to evaluate the cultural relevance of plant species [21].

### Quantitative indices

#### Use value (UV)

To determine the relative importance of the medicinal plants, use value (UV) was used [22].

$${\text{UV}} = {\text{Ui}}/N$$

where Ui is the number of use reports, citations, or mentions by each informant for a particular species, and *N* is the total number of informants who participated in the study. Low numbers signify fewer mentions or citations, whereas high values show a significant volume of use reports or citations from the informants. It counts as one use report or citation each time an informant identifies or describes a species of medicinal plant that is being used to treat a condition or for another reason.

#### Relative frequency citation (RFC)

The relative frequency citation (RFC) is used to determine the relative frequency of reference or mention from the study participants who served as informants and calculated using the formula:

$${\text{RFC}} = {\text{FC}}/N$$

where FC is the number of informants who cited or mentioned plant, and *N* is the total number of informants [23]. The values that are closest to 1 show that nearly all of the informants mentioned a specific medicinal plant that was used to treat a particular illness while low scores show that the usage or purpose of a medicinal plant species is mentioned by few, or occasionally by one, informant.

#### Relative importance (RI)

The relative significance of the medicinal plants according to the use or disease category was ascertained using relative importance (RI) and calculated as follows:

$${\text{RI}} = \left[ {{\text{RFC}}_{(\max )} + {\text{RNU}}_{(\max )} } \right]/2$$

where RFC_(max)_, (RFC_(max)_ = FC/FC_max_) is the RFC of the medicinal plant species and is obtained by dividing the frequency citation of a particular species (FC) by the frequency citation of the species with the highest frequency citation (FC_max_). RNU_(max)_, (RNU_(max)_ = NU/NU_max_) is the relative number of use categories and is obtained by dividing the number of use or disease categories of a particular species (NU) by the number of use categories of the species with the highest use or disease categories (NU_max_) [23]. The high values indicate that a given plant has been used extensively to treat various diseases in several distinct disease categories or use reports whereas low scores indicate that a plant has limited application or function within a small number of disease categories; occasionally, it may only fit into one disease group.

#### Informant consensus factor (ICF)

The informant consensus factor (ICF) was used to assess the homogeneity or degree of agreement of the informants’ knowledge about medicinal plants and calculated as follows:

$${\text{ICF}} = \left( {{\text{Nur}}{-}{\text{Nt}}} \right)/\left( {{\text{Nur}}{-}1} \right)$$

where Nur represents the number of use reports or citations for each illness category, and Nt represents the number of species utilized in that specific category [24].

#### Fidelity level (FL)

The percentage of the most popular and valuable medicinal plant for a specific condition or use category was calculated using fidelity level (FL) using the formula:

$${\text{FL}}\left( \% \right) = {\text{Np}}/N \times 100$$

where NP is the proportion of informants who cited or discussed using a medicinal plant to treat a specific disease category, and *N* is the total number of informants who cited the plant for any other use or purpose [25]. A medicinal plant with a high value will likely have a lot of citations and be the most popular species for treating a specific condition. This ethnobotanical documentation included 14 different use or disease categories that were updated and adapted from the ICD-11 (International Classification of Diseases) for Mortality and Morbidity Statistics [26].

## Results

### Informant demographics

The study included 206 informants, and their information on age, gender, educational level, occupation, and healing experience are shown in Table 1. The majority of informants in the research area were male (62.65%), while 38.35% were female. The age range of informants was 26–35 (12.62%), 36–45 (14.07%), 46–55 (19.90%), and over 66 years (22.82%).

**Table 1:** Demographic characteristics of the informants in the study area

Parameter	Category	Number	Frequency (%)
Sex	Male	127	62.65
	Female	79	38.35
Healing experience	Key informants	59	28.64
	General informants	147	71.36
Age (in years)	26–35	26	12.62
	36–45	29	14.07
	46–55	41	19.90
	56–65	63	31.03
	66 and above	47	22.82
Educational level	Illiterate and elementary school	75	36.41
	High school and diploma	95	46.11
	Graduate and above	36	17.48

When the informants were divided into groups based on gender, a two-tailed independent sample *t*-test showed that there was no statistically significant difference (*p* = 0.79) between males (mean 11.54) and females (mean 8.18) on the documented medicinal plants. When grouped according to educational level and age groups, there was no significant difference in the medicinal plant knowledge with a *p*-value of 0.84 and 0.67, respectively. About 47% of all the informants are 66 years of age or older showing that the rural communities have a clear heritage of openly sharing their traditional knowledge of medicinal plants with the surrounding population, regardless of social class except for education. According to the informants’ levels of education, those with graduate and above (9.29) have the lowest mean for the number of plants mentioned (9.29), followed by an illiterate or elementary education (12.52), and a high school diploma (14.23). With more plants cited in their group than informants with a high school degree, the informants with illiterate and elementary education are all senior individuals with considerable empirical knowledge of medicinal plants. There was a significant difference (*p* = 0.003) in the informants’ knowledge of medicinal plants between those in graduate and above and those in high school, according to the statistical analysis. The results showed that informants with a high school degree had mentioned much more plants than the informants with graduate and above education. On the other hand, no statistically significant variation was observed in the number of plants stated by informants who had completed elementary and high school education (*p* = 0.76) or elementary and graduate above education (*p* = 0.84). The mean education level of high school informants is higher than that of elementary informants, although this difference is not statistically significant. In terms of the mean number of medicinal plants utilized and known in the study area, there was a significant difference (*p* = 0.002) between key and general informants, the key informants had higher levels of knowledge (5.36) than general informants (13.36).

### Diversity and growth form of medicinal plants

A total of 124 medicinal plants from 112 general and 60 plant families were identified as being used by the indigenous people of the Mamit district. With a total of 11 species (8.94%), the Asteraceae family had the most representation and was followed by 9 species (7.31%) of Zingiberaceae, 8 species (6.51%) of Euphorbiaceae, and 7 species (5.69%) of Fabaceae.

**Fig. 3:**
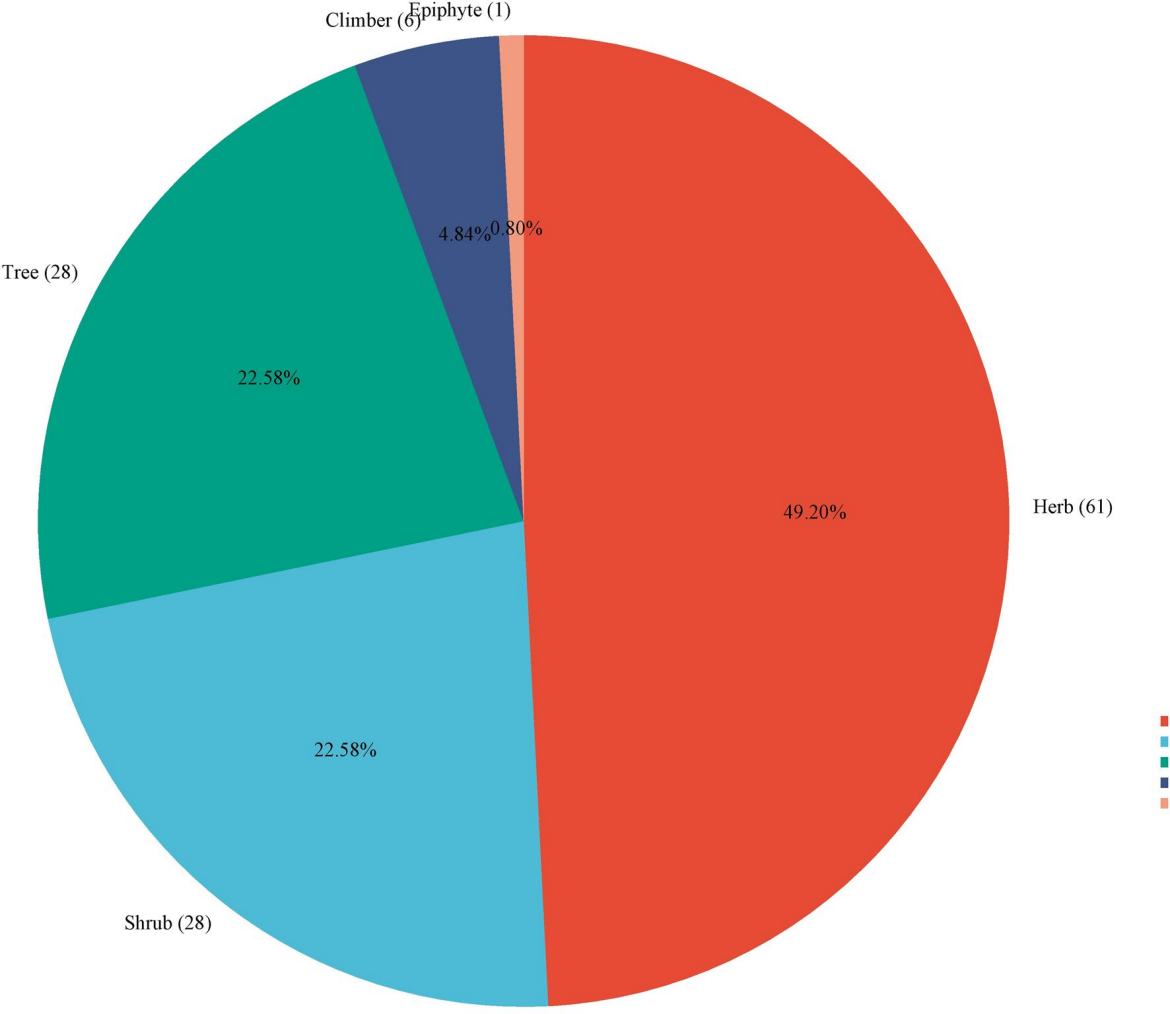
Growth forms of medicinal plant

Herbs, shrubs, trees, climbers, and epiphytes were the preferred growth forms of the therapeutic plants in the study area. With 61 species (49.59%), herbs had the highest percentage of all growth forms, followed by 28 species (22.76) of shrubs and trees, 6 species (4.88%) of climbers, and 1 species (0.81%) of epiphytes (Fig. 3).

### Plant part used, mode of preparation, and administration

**Fig. 4:**
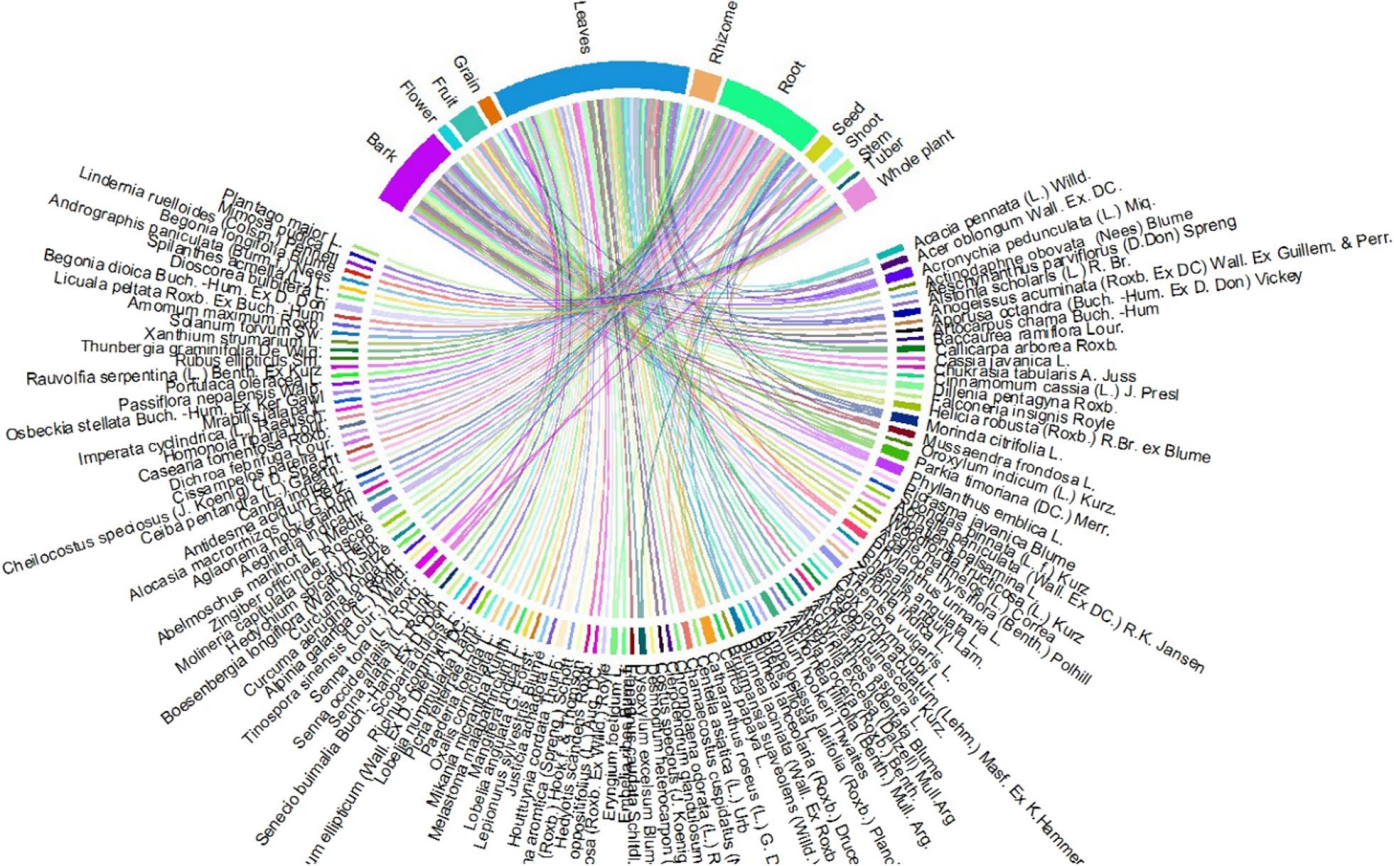
Plant part usage rates

Different plant components such as bark, flowers, fruits, grains, leaves, rhizomes, roots, seeds, stems, tubers, and whole plants are utilized in traditional medicine in the study area. The majority of plant parts that were employed were the leaves from 61 species (49.59%), roots from 31 species (25.20%), and bark from 24 species (19.51%) (Fig. 4).

**Fig. 5:**
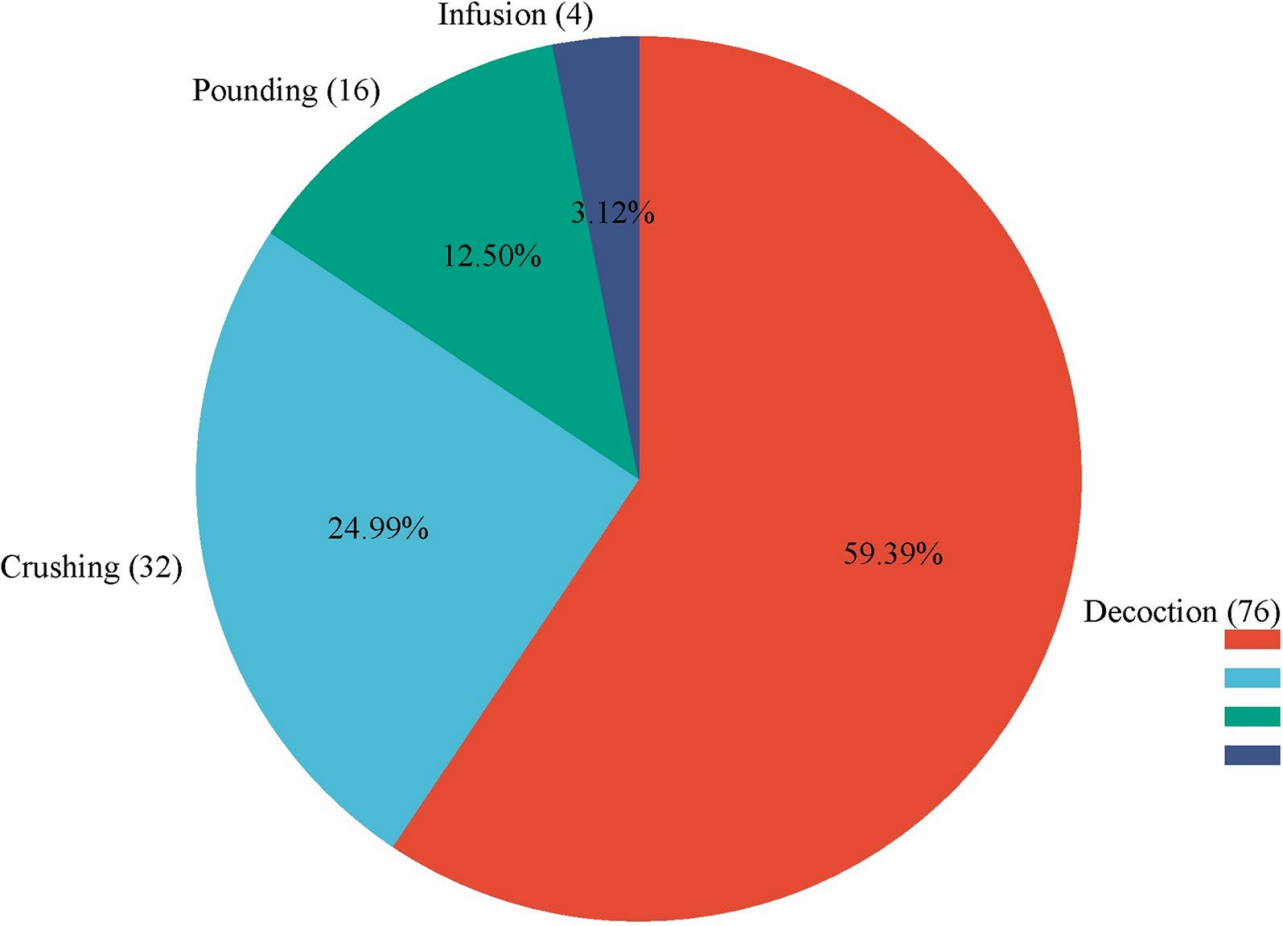
Mode of the preparation of medicinal plants

The indigenous people from the study area concurred that folklore medicines are made using various techniques. Typically, preparation involves decoction from 76 species (61.79%), crushing from 32 species (26.02%), pounding from 16 species (13%), and infusion for 4 species (3.25%) (Fig. 5). In the research area, the majority of traditional medicines (77%) are diluted in water, and 23% are made without the use of any ingredients. Traditional medicines can be delivered orally, topically, by fumigation, or inhalation. Oral uptake (82 species, 66.67%) was the main route of administration in the study area.

### Use value (UV) and relative frequency citation (RFC)

To determine the use value (UV) of the documented medicinal plants, we employed the use report (UR), which gave a way to evaluate their relative importance in the research region and revealed the preferred medicinal plants used by the indigenous people (Table 2). The UV of *Oroxylum indicum* (L.) Kurz*.* and *Curcuma longa* L. was found to be 6.25 and 4.31, respectively, showing the significance of the local practice. Further, *Morinda citrifolia* (3.84), *Cinnamomum cassia* (L.) J. Presl. (3.78), and *Blumea lanceolaria* (Roxb.) (3.26) also revealed the plants with high UV. While, *Licuala peltata* Roxb. Ex Buch.—Hum (0.09) received the lowest recognition for their therapeutic potential. *O. indicum* (0.29)*, A. conyzoides*, and *C. asiatica* (0.28) had the highest RFC values among the documented medicinal plants.

**Table 2:** Medicinal plants used among the indigenous people in the study area

Voucher No.	Species name	Family	Local name	Habit	Part use	Mode of preparation	Medicinal uses	Status	UV	RFC	RI
MZU/BOT/201	*Abelmoschus manihot* (L.) Medik.	Malvaceae	Ui chhu me	Herb	Root and seed	Pounding	Inflammation on tongue (root) and tonsilitis (seed)	LC	0.65	0.15	0.33
MZU/BOT/202	*Acacia pennata* (L.) Willd.	Fabaceae	Khanghu	Shrub	Bark and leaves	Decoction	Bronchitis, asthma, fever, cholera, headache, and snakebites	LC	2.68	0.21	0.49
MZU/BOT/203	*Acacia pruinescens* Kurz.	Mimosaceae	Khang pawl	Shrub	Leaves	Decoction	Asthma, bronchitis, pneumonia, and snakebites	LC	1.13	0.13	0.38
MZU/BOT/204	*Acer oblongum* Wall. Ex. DC	Aceraceae	Thing phingphihlip	Tree	Bark and leaves	Decoction	Fever, stomachache, dysentery, and food poisoning	EN	0.74	0.08	0.29
MZU/BOT/205	*Achyranthes aspera* L.	Amaranthaceae	Bu chhawl	Herb	Leaves	Crushing	Cough, rheumatism, and wounds	NE	0.59	0.09	0.27
MZU/BOT/206	*Achyranthes bidentata* Blume.	Amaranthaceae	Vang vat hlo	Herb	Leaves	Crushing	Menstrual problem (cramps, excessive bleeding)	NE	0.16	0.07	0.16
MZU/BOT/207	*Spilanthes acmella* (L.) L.	Asteraceae	Ankasa pui	Herb	Whole plant	Decoction	Stomachache, headache, and toothache	LC	1.72	0.28	0.59
MZU/BOT/208	*Acmella paniculata* (Wall. Ex DC.) R.K. Jansen	Asteraceae	An sate	Herb	Flower	Crushing	Toothache	LC	0.2	0.09	0.19
MZU/BOT/209	*Acronychia pedunculata* (L.) Miq.	Rutaceae	Par arsi	Tree	Root, bark, and leaves	Decoction	Stomachache, sores, and ulcers	LC	0.48	0.08	0.25
MZU/BOT/210	*Actephila excelsa* (Dalzell) Mull.Arg	Phyllanthaceae	Telengamai	Shrub	Leaves	Crushing	Cuts and urinary problem (urinary tract infection and incontinence)	LC	0.28	0.07	0.16
MZU/BOT/211	*Actinodaphne obovata* (Nees) Blume.	Lauraceae	Pa-khat	Tree	Bark	Decoction	Fractures	LC	0.16	0.07	0.16
MZU/BOT/212	*Aeginetia indica* L.	Orobanchaceae	Sa nghar vaibel	Herb	Root	Pounding	Mumps and inflammation (skin)	NE	0.64	0.16	0.35
MZU/BOT/213	*Aegle marmelos* (L.) Correa.	Rutaceae	Belthei	Tree	Fruit	Crushing	Diabetes, dysentery, and diarrhea	NT	1.12	0.18	0.43
MZU/BOT/214	*Aeschynanthus parviflorus* (D.Don) Spreng	Gesneraceae	Bawlte hlan tai	Epiphytes	Bark	Infusion	Inflammatory (skin)	LC	0.19	0.09	0.19
MZU/BOT/215	*Aganope thyrsiflora* (Benth.) Polhill	Fabaceae	Hulhu	Shrub	Fruit	Decoction	Stomachache and dysentery	LC	0.77	0.18	0.39
MZU/BOT/216	*Ageratum conyzoides* (L.) L	Asteraceae	Vaihlenhlo	Herb	Root and leaves	Decoction	Skin diseases and fever. Roots and Curcuma longa rhizomes boiled with water used for the treatment of gastric cancer	LC	1.76	0.28	0.59
MZU/BOT/217	*Aglaonema hookerianum*	Araceae	Tu bal	Herb	Root	Decoction	Constipation and conjunctivitis	LC	0.24	0.06	0.18
MZU/BOT/218	*Albizia procera* (Roxb.) Benth	Fabaceae	Kangtekpa	Tree	Leaves	Decoction	Ulcers	LC	0.26	0.12	0.24
MZU/BOT/219	*Alchornea tiliifolia* (Benth.) Mull. Arg	Euphorbiaceae	Zawngte nawhlung	Shrub	Leaves	Decoction	Diabetes	LC	0.17	0.08	0.17
MZU/BOT/220	*Allium hookeri* Thwaites	Liliacceae	Mizo purun	Herb	Leaves and root	Pounding	Cancer, viral infection, and inflammatory (redness of skin and body heat)	LC	1.15	0.18	0.43
MZU/BOT/221	*Alocasia macrorrhizos* (L.) G.Don	Araceae	Sai dawl	Herb	Root	Pounding	Inflammation and leprosy	LC	0.25	0.06	0.18
MZU/BOT/222	*Alpinia galanga* (L.) Willd.	Zingiberceae	Ai chal	Herb	Rhizome	Pounding	Stomachache, bronchitis, and rheumatism	LC	0.95	0.15	0.37
MZU/BOT/223	*Alstonia scholaris* (L.) R. Br	Apocynaceae	Thuamriat	Tree	Bark	Pounding	Hypertension, asthma, ringworm, malaria, diarrhea, dysentery, cuts, snakebites, and sores	LC	2.45	0.13	0.27
MZU/BOT/224	*Amomum maximum* Roxb.	Zingiberaceae	Ai du	Herb	Shoot	Decoction	Liver enlargement	LC	0.22	0.11	0.22
MZU/BOT/225	*Ampelocissus latifolia* (Roxb.) Planch	Vitaceae	Vawm hrui	Climber	Leaves	Crushing	Wounds and cuts	LC	0.33	0.08	0.21
MZU/BOT/226	*Andrographis paniculata* (Burm.f.) Nees	Acanthaceae	Hnah khapui	Herb	Whole plant	Decoction	Malaria, stomachache, ulcers, dysentery, diarrhea, snakebites, wounds, liver enlargement, and cholera	LC	2.45	0.13	0.57
MZU/BOT/227	*Anogeissus acuminata* (Roxb. Ex DC) Wall. Ex Guillem. and Perr.	Combretaceae	Zairum	Tree	Bark and leaves	Decoction	Stomachache, fever, diarrhea, chickenpox, measles, hypertension, and burns	LC	3.26	0.22	0.64
MZU/BOT/228	*Antidesma acidum* Retz.	Euphorbiaceae	Thur te an	Shrub	Root	Pounding	Dysentery and diarrhea	LC	0.92	0.22	0.45
MZU/BOT/229	*Aporusa octandra* (Buch.—Hum. Ex D. Don) Vickey	Euphorbiaceae	Chhawntual	Tree	Bark	Decoction	Stomach ulcer, diarrhea, and dysentery	LC	1.14	0.18	0.43
MZU/BOT/230	*Artemisia vulgaris* L.	Asteraceae	Sai	Herb	Root and leaves	Decoction	Fever, asthma, and stomachache	LC	0.59	0.09	0.27
MZU/BOT/231	*Artocarpus chama* Buch.—Hum	Moraceae	Tat kawng	Tree	Bark	Decoction	Inflammatory and diarrhea	LC	0.31	0.07	0.19
MZU/BOT/232	*Baccaurea ramiflora* Lour.	Euphorbiaceae	Pangkai	Tree	Bark	Decoction	Constipation and toothache	LC	0.29	0.06	0.18
MZU/BOT/233	*Begonia dioica* Buch.—Hum. Ex D. Don	Begoniaceae	Se khup thur	Herb	Stem and leaves	Decoction	Dysentery, rash, diarrhea, and sores	LC	0.79	0.09	0.31
MZU/BOT/234	*Begonia longifolia* Blume	Begoniaceae	Sekhup thur hmul	Herb	Whole plant	Infusion	Kidney stone and urinary problem (urinary tract infection)	LC	0.25	0.05	0.16
MZU/BOT/235	*Bidens Pilosa* L.	Asteraceae	Vawkpui thal	Herb	Leaves	Crushing	Skin infection and cancer	LC	0.98	0.24	0.49
MZU/BOT/236	*Blumea lanceolaria* (Roxb.) Druce	Asteraceae	Buarze	Herb	Leaves	Decoction and pounding	Ulcer, asthma, chronic dysentery, skin infection, sores, and scabies	LC	3.26	0.26	1.12
MZU/BOT/237	*Blumea laciniata* (Wall. Ex Roxb.) DC	Asteraceae	Khuanglawr	Herb	Root and leaves	Crushing and pounding	Snakebites	LC	0.27	0.13	0.26
MZU/BOT/238	*Brugmansia suaveolens* (Willd.) Sweet	Solanaceae	Tawtawrawtpar	Shrub	Leaves	Decoction	Asthma	LC	0.26	0.12	0.24
MZU/BOT/239	*Callicarpa arborea* Roxb.	Verbenaceae	Hnah kiah	Tree	Bark and leaves	Decoction	Diabetes, dysentery, diarrhea, ulcers, and cuts	LC	1.09	0.1	0.36
MZU/BOT/240	*Canna indica* L.	Cannaceae	Kungpuimuthi	Herb	Root	Crushing	Fever	LC	0.14	0.06	0.14
MZU/BOT/241	*Carica papaya* L.	Caricaceae	Thingfanghma	Tree	Leaves	Decoction	Jaundice, siabetes, food poisoning fever, and scabies	LC	2.84	0.27	0.66
MZU/BOT/242	*Cassia javanica* L.	Caesalpiniaceae	Makpa zang kang	Tree	Bark	Decoction	Liver enlargement	LC	0.14	0.06	0.14
MZU/BOT/243	*Catharanthus roseus* (L.) G. Don	Apocynaceae	Kumtluang par	Herb	Root, stem, and leaves	Decoction	Cancer, diabetes, dysentery, and diarrhea	LC	1.91	0.23	0.55
MZU/BOT/244	*Ceiba pentandra* (L.) Gaertn	Bombacaceae	Japan pang	Tree	Root	Crushing	Diabetes	LC	0.11	0.05	0.12
MZU/BOT/245	*Centella asiatica* (L.) Urb	Apiaceae	Lambak	Herb	Leaves	Decoction	Diabetes, jaundice, stomachache, hypertension, and skin diseases	LC	2.89	0.28	0.67
MZU/BOT/246	*Chamaecostus cuspidatus* (Nees and Mart) C.D. Specht and D.W. Stev	Costaceae	Sumbul chikhat	Herb	Leaves and root	Decoction	Diabetes	DD	0.21	0.09	0.19
MZU/BOT/247	Cheilocostus speciosus (J. Koenig) C.D. Specht	Zingiberaceae	Sum bul	Herb	Root	Crushing	Kidney failure, fever, jaundice, bronchitis, skin diseases, snakebites, and stomachache	LC	1.29	0.08	0.4
MZU/BOT/248	*Chromolaena odorata* (L.) R.M. King and H. Rob	Asteraceae	Tlangsam	Herb	Leaves	Crushing	Cuts and wounds	LC	1.02	0.24	0.49
MZU/BOT/249	*Chukrasia tabularis* A. Juss	Meliaceae	Zawngtei	Tree	Bark	Infusion and decoction	Cuts, diarrhea, and dysentery	LC	0.57	0.08	0.25
MZU/BOT/250	*Cinnamomum cassia* (L.) J. Presl	Lauraceae	Thakthing	Tree	Bark	Decoction	Diabetes, fever, cancer, kidney failure, heart problem, piles, colic, asthma, headache toothache, and rheumatism	LC	3.78	0.26	0.69
MZU/BOT/251	*Cissampelos pareira* L.	Menispermaceae	Hnahbial hrui	Climber	Root	Pounding	Dysentery, fever, stomach ulcer, urinary problem, cholera, diarrhea, colic, and urinary problem	LC	1.39	0.08	0.44
MZU/BOT/252	*Clerodendrum glandulosum* Lindl.	Verbenaceae	Phuihnam	Tree	Leaves	Decoction	Hypertension	LC	0.62	0.26	0.54
MZU/BOT/253	*Coix lacryma-jobi* L.	Poaceae	Pingpih	Herb	Grains	Decoction	Cancer	LC	0.22	0.1	0.21
MZU/BOT/254	*Costus specious* (J. Koenig) C.D. Specht	Zingiberaceae	Sumbul	Shrub	Leaves	Decoction	Tonsillitis	VU	0.23	0.11	0.22
MZU/BOT/255	*Curcuma aeruginosa* Roxb.	Zingiberaceae	Ailaidumsuak	Herb	Rhizomes	Crushing	Asthma, rheumatic, cough, and anthelmintic	LC	0.66	0.07	0.28
MZU/BOT/256	*Curcuma longa* L.	Zingiberaceae	Ai eng	Herb	Rhizomes	Crushing	Ulcer, dysentery, diarrhea, asthma, cholera, jaundice, bronchitis, and food poisoning	DD	4.31	0.25	0.74
MZU/BOT/257	*Boesenbergia longiflora* (Wall.) Kuntze	Zingiberaceae	Ailaidum	Herb	Rhizome	Infusion	Dysentery and diarrhea	LC	0.74	0.17	0.37
MZU/BOT/258	*Desmodium heterocarpon* (L.) DC	Fabaceae	Berbek	Herb	Leaves	Decoction	Cough	LC	0.24	0.11	0.22
MZU/BOT/259	*Dichroa febrifuga* Lour.	Saxifragaceae	Uitepanganghlo	Shrub	Root	Decoction	Malaria	DD	0.11	0.05	0.12
MZU/BOT/260	*Dillenia pentagyna* Roxb.	Dilleniaceae	Kaih zawl	Tree	Bark and leaves	Decoction	Cancer, dysentery, and asthma	LC	1.43	0.22	0.49
MZU/BOT/261	*Dioscorea bulbifera* L.	Dioscoreaceae	Bachhim	Climber	Tuber	Decoction and pounding	Cancer, asthma, and bronchitis	LC	1.5	0.24	0.53
MZU/BOT/262	*Dysoxylum excelsum* Blume	Meliaceae	Thingthupui	Tree	Leaves and shoot	Decoction	Dysentery, diarrhea, and food poisoning	LC	1.63	0.26	0.56
MZU/BOT/263	*Elaeagnus caudata* Schltdl. Ex Momiy	Elaeagnaceae	Sarzuk	Tree	Leaves	Decoctioon	Stomachache and menstrual problem	LC	0.62	0.15	0.33
MZU/BOT/264	*Embelia ribes* Burm.f	Myrsinaceae	Naufa dawntuai	Shrub	Leaves	Infusion	Jaundice	LC	0.27	0.13	0.29
MZU/BOT/265	*Eryngium foetidum* L.	Apiaceae	Bahkhawr	Herb	Leaves and root	Decoction	Malaria, diabetes, constipation, fever, food poisoning, and anthelmintic	LC	1.61	0.13	0.48
MZU/BOT/266	*Fagopyrum acutatum* (Lehm.) Masf. Ex K.Hammer	Polygonaceae	An bawng	Herb	Grain	Decoction	Diarrhea, colic, and cholera	LC	0.91	0.14	0.38
MZU/BOT/267	*Falconeria insignis* Royle	Euphorbiaceae	Sailutar	Tree	Bark	Crushing	Wounds	DD	0.12	0.05	0.12
MZU/BOT/268	*Flueggea virosa* (Roxb. Ex Willd.) Royle	Euphorbiaceae	Saisiak	Shrub	Leaves	Decoction	Measles, chickenpox, skin diseases, and scabies	LC	1.91	0.22	0.53
MZU/BOT/269	*Glinus oppositifolius* (L.) Aug. DC	Molluginaceae	Bakhate	Herb	Leaves	Decoction and pounding	Fever, inflammation, and wounds	LC	0.76	0.12	0.32
MZU/BOT/270	*Casearia tomentosa* Roxb.	Flacourtiaceae	Vaki thei	Shrub	Root	Decoction	Diabetes	VU	0.14	0.06	0.14
MZU/BOT/271	*Hedychium spicatum* Sm	Zingiberaceae	Aithur	Herb	Rhizome	Decoction	Stomachache, liver problem, inflammation, and snakebites	NT	0.68	0.07	0.28
MZU/BOT/272	*Hedyotis scandens* Roxb.	Rubiaceae	Laikingtuibur	Herb	Leaves	Decoction`	Kidney problems, skin diseases, fever, stomachache, urinary problems, sores, and rheumatism	LC	2.62	0.18	0.58
MZU/BOT/273	*Helicia robusta* (Roxb.) R.Br. ex Blume	Proteaceae	Pasal taka za	Tree	Bark and leaves	Decoction	Ulcers and skin diseases	LC	0.51	0.12	0.28
MZU/BOT/274	*Hodgsonia heteroclita* (Roxb.) Hook. f. and Thomson	Cucurbitaceae	Kha um	Climber	Leaves	Decoction	Ulcer	LC	0.21	0.1	0.21
MZU/BOT/275	*Homalomena aromtica* (Spreng.) Schott	Araceae	Anchiri	Herb	Leaves	Decoction	Stomachache and increase breast milk	LC	0.42	0.09	0.23
MZU/BOT/276	*Homonoia riparia* Lour.	Euphorbiaceae	Tuipuisuhlah	Shrub	Root	Decoction	Stomach ulcers, urinary problems, and gonorrhea	LC	0.33	0.05	0.2
MZU/BOT/277	*Houttuynia cordata* Thunb.	Saururaceae	Uithinthang	Herb	Leaves	Crushing	Viral disease, cancer, and inflammatory	LC	0.99	0.23	0.47
MZU/BOT/278	*Impatiens balsamina* L.	Balsaminaceae	Nuaithang	Herb	Flower	Crushing	Burn and wounds	LC	0.31	0.07	0.19
MZU/BOT/279	*Imperata cyclindrica* (L.) Raeusch	Poaceae	Di	Herb	Root	Crushing	Wounds, dysentery, diarrhea, and anthelmintic	LC	0.58	0.06	0.26
MZU/BOT/280	*Justicia adhadota* L.	Acanthaceae	Kawldai	Shrub	Leaves	Decoction	Asthma, malaria, bronchitis, dysentery, jaundice, and cuts	LC	0.82	0.06	0.34
MZU/BOT/281	*Lepionurus sylvestris* Blume	Olacaceae	An pang thuam	Shrub	Leaves	Decoction	Stomachache, diabetes, and inflammatory	LC	1.61	0.25	0.55
MZU/BOT/282	*Licuala peltata* Roxb. Ex Buch.—Hum	Arecaceae	Lai saw ral	Shrub	Shoot	Decoction	Diarrhea	LC	0.09	0.04	0.1
MZU/BOT/283	*Lindernia ruelloides* (Colsm.) Pennell	Linderniaceae	Tha suih	Herb	Whole plant	Pounding	Wounds, sciatica, and rheumatism	LC	0.95	0.1	0.29
MZU/BOT/284	*Lobelia angulata* G. Forst	Campanulaceae	Choaka thi	Herb	Leaves	Crushing	Diarrhea, stomach ulcer, toothache, and tonsilitis	LC	1.32	0.16	0.43
MZU/BOT/285	*Mangifera indica* L.	Anacardiaceae	Theihai	Tree	Leaves	Decoction	Diabetes, diarrhea, and cancer	LC	1.27	0.2	0.46
MZU/BOT/286	*Melastoma malabathricum* L.	Melastomaceae	Builukham	Shrub	Leaves	Poundin and decoction	Cuts, dysentery, and hypertension	LC	0.82	0.13	0.34
MZU/BOT/287	*Mikania micrantha* Kunth.	Asteraceae	Japan hlo	Herb	Leaves	Crushing	Wounds and cuts	LC	0.89	0.21	0.44
MZU/BOT/288	*Mimosa pudica* L.	Fabaceae	Hlo nuar	Herb	Whole plant	Decoction	Liver problems, kidney stones, fever, ulcers, jaundice, and piles	LC	2.89	0.23	0.63
MZU/BOT/289	*Mirabilis jalapa* L.	Nyctaginaceae	Artuk thuan	Herb	Root	Decoction	Fever, diabetes, and skin disease	LC	0.83	0.13	0.34
MZU/BOT/290	M*olineria capitulata* (Lour.) Herb	Hypoxidaceae	Phaiphak	Herb	Rhizomes	Crushing	Stomachache	LC	0.11	0.05	0.11
MZU/BOT/291	*Morinda citrifolia* L.	Rubiaceae	Noni	Shrub	Root, bark, and leaves	Decoction	Fever, dysentery, asthma, headache, hypertension, diabetes, gastric ulcer, wounds, rheumatism, arthritis, malaria, and menstrual problem	LC	3.84	0.15	0.2
MZU/BOT/292	*Mussaendra frondosa* L.	Rubiaceae	Vakep	Shrub	Bark and leaves	Crushing	Snakebites	LC	0.27	0.12	0.24
MZU/BOT/293	*Oroxylum indicum* (L.) Kurz	Bignoniaceae	Ar chang kawm	Tree	Bark	Decoction	Fever, stomach ulcer, constipation, asthma, dysentery, diarrhea, colic, anthelmintic, rheumatism, inflammation, skin disease, headache, and piles	LC	6.25	0.29	0.89
MZU/BOT/294	*Osbeckia stellata* Buch.—Hum. Ex Ker Gawl	Melastomataceae	Builukhampa	Shrub	Root	Decoction	Stomachache, kidney problem, urinary problem, toothache, anthelmintic, and dysentery	EN	0.91	0.07	0.35
MZU/BOT/295	*Oxalis corniculata* L.	Oxalidaceae	Siakthur	Herb	Leaves	Decoction	Fever, diarrhea, and dysentery	VU	0.33	0.05	0.2
MZU/BOT/296	*Paederia foetida* L.	Rubiaceae	Vawih uih hrui	Herb	Leaves	Crushing	Diarrhea, dysentery, and toothache	LC	0.32	0.04	0.18
MZU/BOT/297	*Parkia timoriana* (DC.) Merr	Fabaceae	Zawngtah	Tree	Bark, fruit, and seed	Decoction	Diarrhea, dysentery, cuts, and menstrual problem	LC	1.31	0.15	0.41
MZU/BOT/298	*Passiflora nepalensis* Walp	Passifloraceae	Nauawimu	Shrub	Root	Decoction	Malaria, dysentery, and hypertension	LC	0.45	0.07	0.24
MZU/BOT/299	*Phyllanthus emblica* L.	Phyllanthaceae	Sunhlu	Tree	Fruit and bark	Crushing	Diabetes, dysentery, diarrhea, and tetanus	LC	1.88	0.22	0.53
MZU/BOT/300	*Phyllanthus urinaria* L.	Phyllanthaceae	Mitthi sunhlu	Herb	Fruit and leaves	Decoction	Urinary problems (urinary tract infection), dysentery, fever, liver problems, jaundice, diabetes, cholera bronchitis, leprosy, anemia, and asthma	DD	2.29	0.09	0.58
MZU/BOT/301	*Physalis angulata* L.	Solanaceae	Chalpangpuak	Herb	Fruit	Crushing	Diabetes, toothache, and inflammation	LC	0.45	0.07	0.24
MZU/BOT/302	*Picrasma javanica* Blume	Simarubaceae	Thingdamdawi	Tree	Bark	Infusion	Fever, hypertension, stomachache, and dysentery	LC	0.37	0.04	0.22
MZU/BOT/303	Picria felterrae Lour.	Linderniaceae	Khatual	Herb	Leaves	Decoction	Liver enlargement, fever, and stomachache	LC	0.47	0.07	0.24
MZU/BOT/304	*Plantago major* L.	Plantaginaceae	Kelba an	Herb	Whole plant	Decoction	Malaria, diabetes, chronic ulcers, and wounds	LC	1.31	0.15	0.41
MZU/BOT/305	*Portulaca oleracea* L.	Portulaccaceae	Hmutau	Herb	Root	Decoction	Dysentery and diarrhea	LC	0.23	0.05	0.16
MZU/BOT/306	*Lobelia nummularia* Lam	Campanulaceae	Choakthi	Herb	Leaf	Crushing	Dysentery	LC	0.16	0.07	0.16
MZU/BOT/307	*Rauvolfia serpentina* (L.) Benth. Ex Kurz	Apocynaceae	Rulturzung	Herb	Root	Decoction	Stomachache, hypertension, snakebites, epilepsy, and anthelmintic	LC	0.39	0.03	0.24
MZU/BOT/308	*Rhynchotechum ellipticum* (Wall. Ex D. Dietr.) A. DC	Gesneraceae	Tiar rep	Herb	Leaves	Decoction	Cancer	LC	0.17	0.07	0.16
MZU/BOT/309	*Ricinus communis* L.	Euphorbiaceae	Mutih	Shrub	Leaves	Decoction	Stomach ulcers and sciatica	LC	0.38	0.08	0.21
MZU/BOT/310	*Rubus ellipticus* Sm	Rosaceae	Hmutau	Shrub	Root	Decoction	Diarrhea and dysentery	LC	0.23	0.05	0.16
MZU/BOT/311	*Scoparia dulcis* L.	Plantaginaceae	Perhpawng chaw	Herb	Leaves	Crushing	Kidney stones, diabetes, diarrhea, dysentery, toothache, cuts, burns, snakebites, and stomachache	LC	0.92	0.04	0.41
MZU/BOT/312	*Senecio buimalia* Buch.—Ham. Ex D. Don	Asteraceae	Saiekhlo	Shrub	Leaves	Decoction	Stomachache, cancer, dysentery, and hypertension	LC	1.53	0.18	0.47
MZU/BOT/313	*Senna alata* (L.) Roxb	Caesalpiniaceae	Dadu hlo	Shrub	Leaves	Crushing	Snakebites, eczema, ringworm, and gonorrhea	LC	0.35	0.03	0.21
MZU/BOT/314	*Senna occidentalis* (L.) Link	Caesalpiniaceae	Reng an	Herb	Leaves	Decoction	Fever, liver problems, bronchitis, hypertension, menstrual problem, malaria, and anthelmintic	LC	1.19	0.07	0.39
MZU/BOT/315	*Senna tora* (L.) Roxb.	Caesalpiniceae	Kel be	Herb	Root and leaves	Crushing	Skin diseases	LC	0.14	0.06	0.14
MZU/BOT/316	*Solanum anguivi* Lam	Solanaceae	Tawkte	Shrub	Fruit	Decoction and crushing	Asthma, fever, and hypertension	LC	1.63	0.26	0.56
MZU/BOT/317	*Solanum torvum* Sw	Solanaceae	Tawkpui	Shrub	Seed	Crushing	Toothache	LC	0.32	0.15	0.29
MZU/BOT/318	*Spondias pinnata* (L. f.) Kurz	Anacardiaceae	Tawitaw	Tree	Bark	Decoction	Diarrhea, dysentery, rheumatism, and cuts	LC	0.69	0.08	0.29
MZU/BOT/319	*Thunbergia graminifolia* De Wild	Acanthaceae	Vako	Herb	Root	Pounding	Kidney stones and jaundice	NT	0.25	0.05	0.16
MZU/BOT/320	*Tinospora sinensis* (Lour.) Merr	Menispermaceae	Hrui vankai	Climber	Stem and leaves	Decoction	Cancer, malaria, and diabetes	DD	1.28	0.2	0.46
MZU/BOT/321	*Woodfordia fructicosa* (L.) Kurz	Lythraceae	Ainawn	Shrub	Flower	Pounding	Ulcers and skin infection	LC	0.31	0.07	0.19
MZU/BOT/322	*Xanthium strumarium* L.	Asteraceae	Chabet	Herb	Root	Decoction	Fever, cancer, and urinary problem	LC	0.35	0.05	0.2
MZU/BOT/323	*Zanonia indica* L.	Cucurbitaceae	Lalruanga dawibur	Climber	Fruit	Decoction	Stomachache	LC	0.48	0.22	0.41
MZU/BOT/324	*Zingiber officinale* Roscoe	Zingiberaceae	Sawhthing	Herb	Rhizome	Pounding	Asthma, rheumatic, anemia, and inflammatory	LC	2.09	0.25	0.59

### Relative importance index (RI)

The number of uses for each medicinal plant by use or disease category was ascertained using RI. *B. lanceolaria* (1.12) had the highest RI value followed by *O. indicum* (0.89) and *C. longa* (0.74), respectively. The RI results were also comparable with the result of UV values (Table 2).

### Informant consensus factor (ICF)

**Fig. 6:**
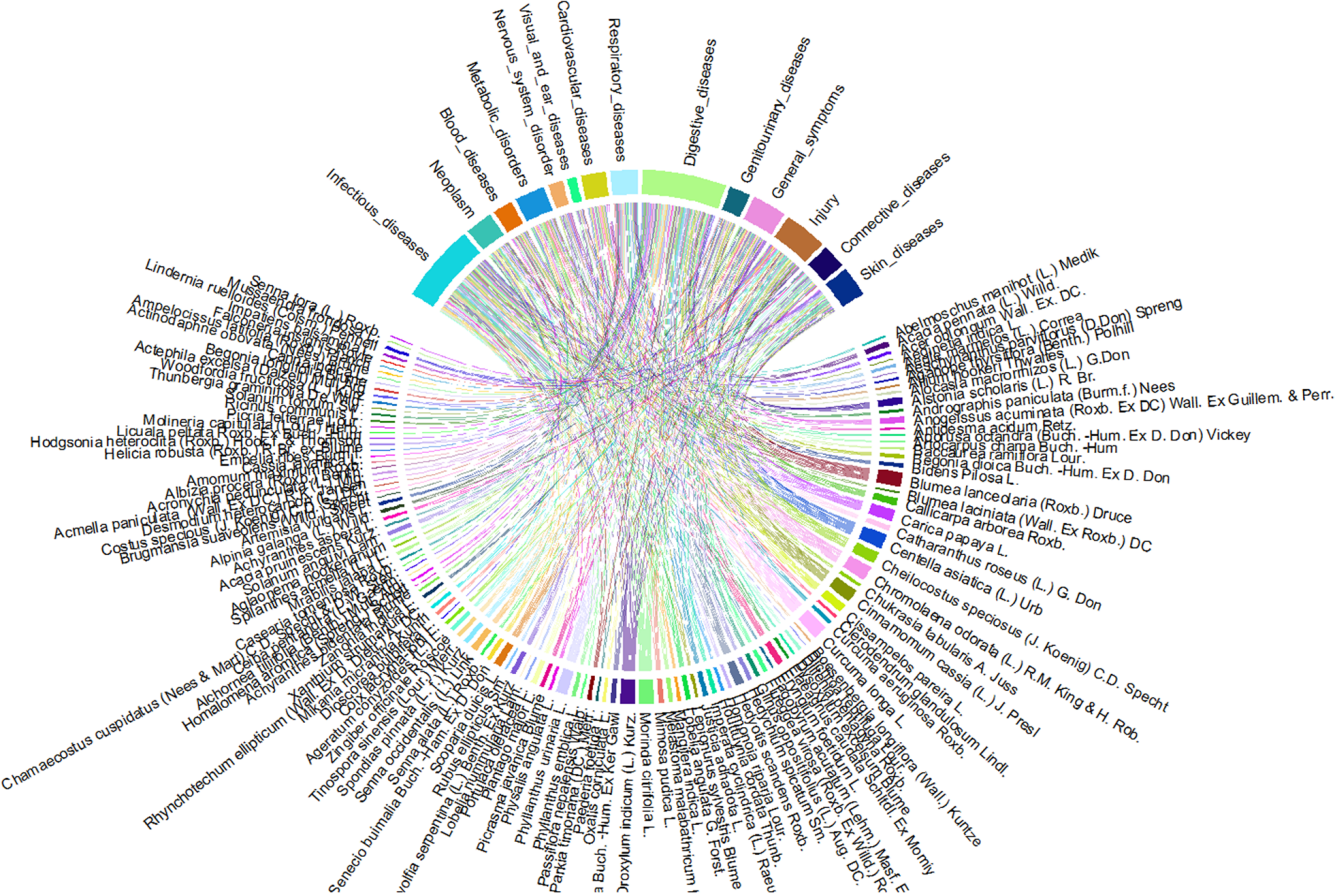
Analysis of informant consensus factor from the study area

The culturally significant medicinal plants utilized by various informants within the same use or ailment category were evaluated using ICF. Under 14 different disease categories, the present study listed 54 diseases (Table 3 and Fig. 6). The cardiovascular diseases category (heart disease and hypertension) had the highest ICF score (0.94), and *C. glandulosum* was the most popular taxon of medicinal plants. While the general symptoms had the lowest ICF score (0.71), the listed diseases included body pain, hemorrhage, and fever.

**Table 3:** Ailment category, disease under each category, number of plants used, used report, ICF, FL, most cited taxa, and purpose of most cited taxa

Ailment category	Disease under each category	Number of plants used	Use report	ICF	FL (%)	Most cited taxa	Purpose of most cited taxa
Infectious diseases	Amoebiasis, chickenpox, measles, mumps, tetanus, tuberculosis, typhoid, rabies, dysentery, and inflammation cholera	55	337	0.83	76	*Aeschynanthus parviflorus*	Dysentery
Neoplasms	Cancer	12	97	0.88	39	*Catharanthus roseus*	Cancer
Blood diseases	Anemia and menstrual problem	6	31	0.83	40	*Achyranthes bidentata*	Menstrual problem
Metabolic diseases	Diabetes	19	101	0.82	91	*Lepionurus sylvestris*	Diabetes
Nervous system disorder	Convulsion, headache, paralyzes, and dizziness	5	21	0.8	72	*Spilanthes acmella*	Headache
Visual and ear diseases	Conjunctivitis and ear problem	2	11	0.9	44	*Aglaonema hookerianum*	Conjunctivitis
Cardiovascular diseases	Heart disease and hypertension	13	201	0.94	63	*Clerodendrum glandulosum*	Hypertension
Respiratory diseases	Asthma, cough, lung problem, pneumonia, tonsilitis, and bronchitis	21	101	0.8	38	*Brugmansia suaveolens*	Asthma
Digestive diseases	Abdominal pain, stomachache, constipation, diarrhea, gallbladder problem, hepatitis, indigestion, liver disease, stomach ulcer, toothache, and jaundice	67	443	0.85	87	*Curcuma longa*	Diarrhea
Skin diseases	Rash, eczema, and skin problem	12	112	0.9	55	*Ageratum conyzoides*	Skin problem
Genitourinary diseases	Kidney stone and urinary tract problem	11	49	0.79	63	*Hedyotis scandens*	Kidney stone
Connective diseases	Muscle swelling, rheumatoid, arthritis, and sciatica	9	46	0.82	70	*Lindernia ruelloides*	Rheumatoid
General symptoms	Body pain, hemorrhage, and fever	23	78	0.71	82	*Oroxylum indicum*	Fever
Injury	Burns, cuts, wounds, insect bites, and fracture	16	82	0.81	77	*Chromolaena odorata*	Cuts

### Fidelity level (FL)

The relative significance of a medicinal plant species within each ailment or usage category was assessed using FL. The medicinal plants with the highest FL values were *Lepionurus sylvestris* (91%) and *Curcuma longa* (87%), respectively (Table 3). Regarding ailments and symptoms or signs affecting metabolic diseases, *L. sylvestris* is the medicinal plant that is most frequently prescribed for the treatment of diabetes. *C. longa* is the most frequently mentioned and chosen species for treating diarrhea under the digestive system disease ailment category. Under the respiratory ailment category (asthma), *Brugmansia suaveolens* has the lowest FL value among the medicinal plants.

### Comparison between various quantitative indices

Table 4 shows the ranking of medicinal plants with the most disease or use categories, UV, RFC, and RI values. Medicinal plants with the highest UV, RFC, and RI values are the most highly regarded and culturally significant in the study area. The number of use reports, the frequency of citations from the informants, and various applications or purposes in ailment categories are used to evaluate them. In all three indices, the top 10 medicinal plants were nearly identical, with the possible exceptions of *Andrographis paniculata, Alstonia scholaris*, and *Phyllanthes urinaria* being listed in the NU (number of use). These three plants have a high number of multiple uses; however, their UV, FC, and RI are not high compared to the other species. A specific medicinal plant is assumed to have a low-use report, frequency citation, and disease category if its UV, RFC, and RI values are low. The current investigation also showed similarities between the UV, RFC, and RI values of some reported species. Nonetheless, each index yields a different rating for each species. Based on both RFC and UV indices, *O. indicum* is ranked one. This could be because the species is the most well-known plant in the majority of research sites and is frequently referenced by informants. It has been proposed that UV value, rather than citation count, is a more accurate indicator of use diversity [27]. In line with this, the species in our study that is most frequently used determine the UV value rather than those that are cited by more informants. While *B. lanceolaria* ranks as one and *O. indicum* ranked as two in the RI index. The degree of diversity in medical applications determines the RI. The plant's high level of diversity and use for the treatment of various ailments could be the basis for its top ranking in RI. Without taking into account the number of informants or the likelihood of being assigned to one of the categories, the RI was designed as a broad indicator of the diversity of uses. It is based on the adaptability of use categories as well as more specialized uses. However, the UV and RFC provide the average number of use reports per informant and species. This must be the cause of the disparity in the rankings of RI and UV and RFC. In addition, a considerable positive correlation was observed between the relative importance of plant use and the local relevance of each medicinal plant, as indicated by the Pearson correlation coefficient of 0.69 (*p* < 0.003) between RFC and UV. This finding was in comparison with earlier research that also found a strong positive association between RFC and UV [28, 29]. Further, a correlation between RFC and RI showed a negative correlation for the plant species (*r*^2^ = − 0.42, *p* < 0.024), suggesting that their patterns were specific to species origin. According to the participant consensus, utility in treating illnesses, and species origin, we conclude that RFC and RI are independent. Based on the informants’ citations, high UV, and various applications across many disease categories, B*. lanceolaria, C. longa,* and *O. indicum* are the most culturally favored, valued, advised, and significant medicinal plants among the indigenous people.

**Table 4:** Medicinal plants with the highest number of disease use (NU), UV, RFC, and RI

Rank	Plant	NU	Plant	UV	Plant	RFC	Plant	RI
1	*Oroxylum indicum*	13	*Oroxylum indicum*	6.25	*Oroxylum indicum*	0.29	*Blumea lanceolaria*	1.12
2	*Morinda citrifolia*	12	*Curcuma longa*	4.31	*Centella asiatica*	0.28	*Oroxylum indicum*	0.89
3	*Phyllanthes urinaria*	11	*Morinda citrifolia*	3.84	*Ageratum conyzoides*	0.28	*Curcuma longa*	0.74
4	*Cinnamomum cassia*	10	*Cinnamomum cassia*	3.78	*Spilanthes acmella*	0.28	*Cinnamomum cassia*	0.69
5	*Ageratum conyzoides*	9	*Blumea lanceolaria*	3.26	*Carica papaya*	0.27	*Centella asiatica*	0.67
6	*Alstonia scholaris*	9	*Ageratum conyzoides*	3.26	*Blumea lanceolaria*	0.26	*Carica papaya*	0.66
7	*Andrographis paniculata*	9	*Centella asiatica*	2.89	*Clerodendrum glandulosum*	0.26	*Morinda citrifolia*	0.64
8	*Curcuma longa*	8	*Mimosa pudica*	2.89	*Cinnamomum cassia*	0.26	*Mimosa pudica*	0.63
9	*Blumea lanceolaria*	8	*Lepionurus sylvestris*	2.68	*Curcuma longa*	0.25	*Spilanthes acmella*	0.59
10	*Centella asiatica*	7	*Clerodendrum glandulosum*	2.62	*Lepionurus sylvestris*	0.25	*Ageratum conyzoides*	0.59

## Discussion

The fact that traditional healers typically prefer to impart their knowledge of native medicinal plants to other men may account for the high proportion of male informants in the research area. Similarly, results from other studies from the states revealed the preponderance of men [30, 31].

The dominant families used were Asteraceae and Fabaceae, which might be due to the stronger adaptation potential of the species in these families over a wider range of elevations. Similar results were also reported from the previous work [32, 33].

Herbs are the most commonly reported plant species. This could be owing to the local people’s ease of access and abundance. Similarly, most ethnobotanical research in Mizoram [30] and other countries [33, 34] also reported the dominant uses of herbs for traditional medicine.

The majority of medicinal plant components used to treat any health issues are leaves, which is consistent with research from the states and other countries [33, 35]. The plant part known as leaves is easily obtainable and always available during emergencies, particularly in tropical nations like India. Due to their ease of growth and regeneration, leaves are a more sustainable crop to harvest than other plant parts.

The indigenous people of the study area have evolved knowledge of remedy dosing based on their historical and long-term practical experience with employing traditional medicinal plants for various diseases. The common method of dosage delivery was based on the severity of the ailments treated, the health status of the patient, age, and the experience of the local healer administering the remedy, even though there were differences in dosage units and administration periods. The amount or dose of the remedy was measured using various instruments such as tablespoons, tea cups, etc.

The mean number of medicinal plants cited by male and female informants in the study area did not differ significantly. This demonstrates that all family members have the same level of expertise and that both men and women are in charge of providing primary health care. Similar studies from the previous reports also showed insignificant medicinal plant knowledge between male and female informants [34, 36]. Key informants were predictably more knowledgeable than general informants which was also reported from the previous work [37]. This may be explained by their extensive experience and extreme discretion when employing therapeutic plants.

Among the known medicinal plants, *O. indicum* exhibited the highest UV and RFC values, indicating that it is the most valued and favored medicinal plant for treating various diseases across several ailment categories. The phytochemical compound, Baicalein, which serves as a major component of *O. indicum* has various biological potentials such as anticancer, antibacterial, anti-hyperglycemia, neurogenesis, cardioprotective, anti-adipogenesis, anti-inflammatory, and wound healing [38]. *B. lanceolaria* has the highest RI value among the documented medicinal plants. The plant has various biological activities such as anticancer, anti-inflammatory, and antimicrobial potentials [39].

The best agreement among the informants about the usage of medicinal plant species for treating cardiovascular diseases (0.94) is indicated by the highest reported ICF value in the study area. There may be a high incidence of the classified diseases based on the highest informants’ agreement and high-use report for this categorizing of diseases. Heinrich et al. [18] stated that finding species with a higher likelihood of possessing intriguing bioactive components requires a high ICF value. When choosing which species to preserve in an environment where medicinal plant species are steadily disappearing, a high informant consensus factor is also a crucial consideration.

The plants *L. sylvestris* (91%) and *C. longa* (87%) have the highest FL values against diabetes and diarrhea, respectively. These results may indicate that the respective plants have a strong healing potential. Studies using phytoextraction to demonstrate the effectiveness of bioactive components can benefit from using plants with high FL values.

The main challenge to the conservation and management of natural products in Mizoram is the unsustainable harvesting of natural products, particularly medicinal plants. In the present study, 124 documented medicinal plant species face varying categories of threat based on IUCN criteria and local perspective. Two species are classified as endangered, three species as near threatened and vulnerable, respectively, five species are classified as data deficient, 107 species are considered as least concern, and three species have not been evaluated. Overharvesting as a result of trade pressure is the main issue. Some species are also disappearing because of habitat degradation, cattle grazing, forest fires, etc. Our research indicates that the majority of the medicinal plants that have been documented pose the least threat to conservation, meaning that most of the plants are not in danger of being lost.

### Health significance of the present study for the indigenous community

The most prevalent medical problems in the studied area are listed in Table 3. In our study, medicinal plants that are used to treat each of these illnesses are extensively represented. Digestive diseases such as diarrhea, stomachache, etc., and metabolic and cardiovascular diseases such as diabetes, hypertension, etc., can cause considerable harm to public health in the community. Given that these diseases often affect populations that are already at risk, we would want to emphasize the significance of the treatment available for treating these diseases and their aftereffects. According to the World Health Organization [40], diarrhea has been the second leading cause of death and a major public health concern in low-income countries and developing countries like India. The majority of the current research region is made up of isolated areas without adequate water supplies, forcing the locals to rely on water from wells, rivers, and other sources. The most common illness in the area under study is diarrhea. Due to its frequent occurrence in isolated rural areas and potential threat to life due to lack of access to conventional medical care in the critical period of infection, the Ministry of Health and Family Welfare lists various common health issues in the states, but diarrhea is not one of them because the risk is quite low in metropolitan areas with access to medical care. Nonetheless, in isolated rural communities, it frequently poses a life-threatening problem. In addition, diarrhea is the most frequent pediatric illness and a major contributor to infant and child mortality [41, 42]. Similar findings were also observed in neighboring countries [33, 43] and other developing countries such as South Africa [44], Uganda [45], and Zimbabwe [46]. Similar to other countries [43, 44], stomachaches are likewise the most common health issue in the research area. Malnutrition and improper food handling are probably the cause of stomach problems. Traditionally, people have used medicinal plants to treat certain illnesses, and they have been quite successful in many circumstances. Because traditional medicine is practiced by the locals as the primary health-care system and prevalent health conditions are taken into consideration when formulating health policies, the current findings offer crucial insights for health officials. According to the WHO [40], the number of adults with hypertension has increased largely in low- and middle-income countries, among non-communicable diseases, cardiovascular diseases are responsible for annual deaths of 17.9 million, followed by cancer (9 million deaths), respiratory diseases (3.9 million deaths), and diabetes (1.6 million deaths). Both medication and lifestyle modifications are part of the treatment for hypertension. Reducing alcohol and tobacco use, improving nutrition and exercise, etc., are examples of lifestyle modifications [40]. Given the poor income and high prevalence of tobacco use in the research areas, hypertension is regarded as the most common ailment in the population. The indigenous people treat hypertension mainly with preparation from *C. glandulosum* leaves.

The principal contribution of our research is the documentation of traditional knowledge about how to treat each of the aforementioned illnesses, which are common in the community of the study area. It contributed to the preservation of the conservation of biodiversity and education of allopathic medical professionals about traditional medicine, in addition to improving public health, particularly in remote areas.

### Comparison with the previous ethnobotanical studies

While several ethnobotanical and ethnomedicinal investigations have been carried out in India, very few of these research have included quantitative analysis. While a small number of researches concentrated on particular indigenous people or tribal communities, the majority of ethnobotanical studies purposefully selected key informants who were only knowledgeable about medicinal plants, such as residents, traditional healers, herbalists, and elders.

Despite having rich diversity and indigenous cultural groups, Mizoram is the least well-documented among different states of India. In Mizoram, three indigenous groups were documented, namely, the Mizo tribe, the Mara, and the Chakma tribe in the eastern (Champhai) region and southern region of Mizoram, respectively [13–18, 30, 31, 47]. In the northeastern region of India, the ethnomedicinal uses have been documented from the Adi, Apatani, Bangni, Chakma, Hill Miri, Khamti, Minyong, Mishmi, Monpa, and Nyshi communities from Arunachal Pradesh [48, 49], the Mikir, Mishing, Deori, Dimasa, Rajbangshi, Hmar, Soantali, and Tai-Ahom tribes from Assam [48], the Kabui, Mao, Meitei, Paite, Tangkhul-Naga, Thadou, and Zou tribes from Manipur [50], the Khasis, Jaintias, and Garos tribes from Meghalaya [51], the Angami, Ao, Konyak, Lotha, Phom, Rengma, Sangtam, Sumi, Yamchunger, and Zeliang tribes from Nagaland [52], the Lepcha tribe from Sikkim [48], and the Bengali, Darlong, Halam, Reang, and Tripuri tribes from Tripura [48], respectively. In Rajasthan, the plant utilization among various ethnic groups such as Bhil, Bhil-Meena, Damor, Dhanka, Garasia, Kathodi, Kokna, Kolidhor, Naikara, Pateilia, Meena, and Seharia has been documented [53]. Among the region of Central India, ethnomedicinal plants have been documented from various parts such as Datia, Tikamgarh, Chhatarpur, Panna, Sagar, and Damoh, in Madhya Pradesh [54].

A previous work from Manipur has also reported *O. indicum* as a potential anticancer medicinal plant which is comparable to the present study [55]. The medicinal plant *C. glandulosum* has been reported as the folk remedy for the treatment of hypertension in four research works published from different states of the northeastern regions of India [48], and *C. longa* has also been reported as the most relevant species using quantitative indices [31] similar to the present study.

The inclusion of data on adverse or side effects in this study presents more extensive documentation of ethnopharmacology in the western region of Mizoram, which may serve as a resource for ethnomedical, biological, and pharmacological research in the future.

### Conservation status

The International Union for Conservation of Nature (IUCN) Red List of 124 documented medicinal plants includes 3 species (2.42%) that have not been evaluated (NE) or have a result, 107 species (86.29%) that are considered to be of least concern (LC), 2 species (1.63%) that are endangered (EN), 3 species (2.42%) that are near threatened (NT) and vulnerable (VU), and 5 species (4.03%) that lack sufficient information (DD) (IUCN 2022). Based on our research, and documentation of the status of medicinal plants based on State Medicinal Plant Board Mizoram, the majority of known medicinal plants offer the least threat to conservation, which means that there is little chance for the plants going extinct.

### Threats to traditional medicinal knowledge and medicinal plants

The majority of the people who were familiar with the traditional medicine from the study area, according to the informants’ interview results, were between the age range of 30 and 60, and those in this age range also showed a higher level of descriptive knowledge of medicinal plants. The legacy of traditional medicine can take several forms. It can be passed down through families, through self-directed learning, through hands-on training, through knowledge accumulation, or through gathering medicinal formulations. The origin and details of therapeutic practices are unknown due to the lack of a written tradition. Utilization of traditional medicine has decreased in the studied areas as a result of modern medicine's easy accessibility. Further, changes in socioeconomic conditions may result in the extinction or reduction of medicinal plants and accompanying indigenous knowledge. In addition, human endeavors such as areca nut and rubber plantations underforested economy have led to a startling decline in the region’s biodiversity. The fall in knowledge and cultivation practices, along with wild plant harvesting, have resulted in a drop in the availability of medicinal plants within the community. The interaction of cultural, historical, environmental, and belief systems has had a significant impact on the development and evolution of traditional medical knowledge among the indigenous people. These societies place great importance on traditional knowledge, viewing it as a deeply ingrained cultural legacy. Traditional medicine is more than just a means of treatment for the indigenous people, it is a symbol of cultural identity. The results highlight how crucial it is to maintain and advance traditional knowledge of medicinal plants to safeguard cultural heritage and advance sustainable development. A crucial component of preserving and advancing minority cultures is the transmission of traditional medicinal knowledge. The government, academics, communities, and local healers are among the many parties collaborating to save the traditional medicine culture that is in jeopardy. These collaborative efforts are aimed at documenting and preserving traditional knowledge, offering training and education to local healers and younger generations, and developing plans for the future growth of this valued information [56].

## Conclusions

The rural populations’ traditional medicine practices are heavily impacted by their experiences, as well as the culture that has been passed down through generations through oral communication. Indigenous medicinal practices are becoming more susceptible as modern medicine slowly seeps into remote areas. Folkloric practices are steadily vanishing due to a lack of interest in the younger generation and the availability of non-prescription medications. It is important to appropriately document the experiences of senior citizens and the elderly whose empirical knowledge of the use of medicinal plants in ethnomedicine is acknowledged and treasured. Their wealth of traditional knowledge has been documented, and this documentation has produced new insights and expanded the range of treatments available for various illnesses.

The region boasts a notable diversity of medicinal plants, although intensive farming occupies the majority of the land. Most of these medicinal plants are not classified, appraised, or listed as least concern on the IUCN Red List. Plants have many uses for humans and provide enormous promise for the development of new drugs. As such, the findings of this ethnobotanical study will form the foundation for more pharmacological research, particularly with the most frequently mentioned, favored, esteemed, and significant therapeutic plants. The preservation of medicinal plants will also be brought to light, providing a secure and efficient substitute that can be integrated into primary health-care services.

## Data Availability

All data generated or analyzed during this study are included in the manuscript.

## Abbreviations

ICF: Informant consensus factor
UV: Use value
FL: Fidelity level
RFC: Relative frequency citation
RI: Relative importance index
IHEC: Institution Human Ethical Committee
MS Excel: Microsoft Excel
ICD-11: International Classification of Diseases
IUCN: Union for Conservation of Nature
NE: Not evaluated
LC: Least concern
EN: Endangered
NT: Near threatened
VU: Vulnerable
DD: Data deficient
NU: Number of use
